# Hyperviscosity encephalopathy in suspected polycythemia vera at high altitude: a case report

**DOI:** 10.3389/fmed.2026.1891683

**Published:** 2026-07-23

**Authors:** Shaoli Yao, Xiaorong Chen

**Affiliations:** Department of Neurology, Hospital of Chengdu Office of The People's Government of Xizang Autonomous Region, Chengdu, Sichuan, China

**Keywords:** cerebral infarction, erythropoietin, high-altitude, hyperviscosity syndrome, polycythemia vera

## Abstract

**Background:**

Polycythemia vera (PV) is a JAK2-associated clonal myeloproliferative neoplasm characterized by erythroid hyperplasia. To the best of our knowledge, this is the first reported case of acute hyper-viscosity encephalopathy as the initial manifestation of suspected PV in a resident of extreme high altitude—a diagnosis frequently mistaken for altitude-induced secondary polycythemia in this population.

**Case report:**

A 37-year-old Tibetan man, lifelong resident at 4,100 m, presented with acute encephalopathy characterized by altered consciousness, gait ataxia, and headache. Neurological examination confirmed higher cortical dysfunction. Laboratory studies showed marked erythrocytosis (hemoglobin 229 g/L, hematocrit 77.4%) with paradoxically suppressed serum erythropoietin (EPO, 1.2 mIU/mL). Brain magnetic resonance imaging (MRI) with diffusion-weighted imaging (DWI) revealed acute bilateral frontoparietal and centrum semiovale diffusion-restricted lesions. Neurological symptoms resolved rapidly after osmotic therapy for intracranial hypertension. These findings were consistent with suspected PV based on WHO diagnostic criteria.

**Conclusion:**

This case suggests that acute infection may trigger life-threatening hyper-viscosity syndrome in suspected PV at extreme altitudes. EPO assessment and JAK2 mutational analysis are recommended to be included in the diagnostic algorithm to distinguish malignant PV clones from compensatory polycythemia and to prevent catastrophic neurological outcomes in high-altitude residents.

## Introduction

Polycythemia vera (PV) is a JAK2-mutated clonal myeloproliferative neoplasm (1) that drives pathological erythroid hyperplasia and carries a high risk of thrombotic complications. Cerebrovascular events—including cerebral infarction (2, 3), cerebral venous thrombosis (4–6), and hemorrhagic (7)—are major contributors to disease burden. In high-altitude populations, where physiological erythrocytosis is common, PV often goes undetected until a catastrophic event occurs (8). This diagnostic delay is concerning given the difference in malignant potential between PV and adaptive polycythemia. The present case illustrates this diagnostic challenge by describing acute hyper-viscosity encephalopathy as the initial presentation of suspected PV at high altitude. This work aims to define: (1) a potential trigger of acute decompensation observed in this case; (2) key diagnostic indicators that differentiate malignant PV from physiological adaptation; and (3) altitude-specific interventions to prevent life-threatening complications.

## Case report

A 37-year-old Tibetan man with lifelong residence at 4,100 meters presented to our emergency department with a 3-day history of progressive confusion, gait instability, and throbbing headache. He had experienced an upper respiratory tract infection 1 week before symptom onset (diagnosed clinically and radiographically; microbiological confirmation was not obtained). His medical history was unremarkable for hypertension, diabetes, cardiovascular diseases, thrombotic events, tobacco use, or significant alcohol consumption.

**Figure 1:**
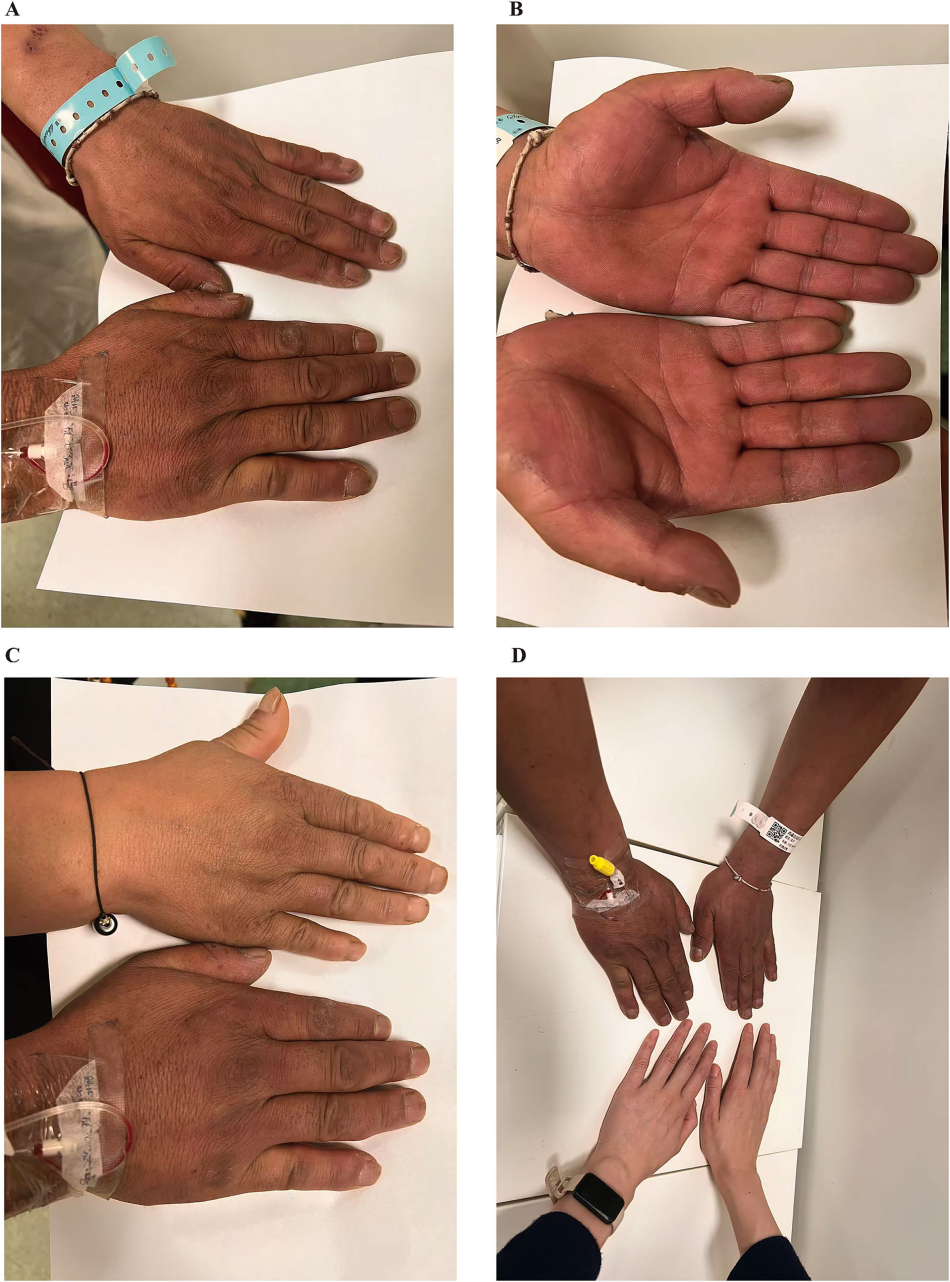
Comparative characterization of hand morphology: index patient, cohabiting high-altitude relative, and lowland residents. **(A)** Dorsal view of the patient’s hands. **(B)** Palmar view of the patient’s hands. **(C)** Comparative view of the patient’s hands and those of a first-degree relative (sister) residing at high altitude (upper panel: sister’s hands; lower panel: patient’s hands). **(D)** Comparative view of the patient’s hands versus those of a representative lowland resident (upper panel: patient’s hands; lower panel: lowland resident’s hands).

On admission, peripheral oxygen saturation (SpO_2_) was 93% on room air. Arterial blood gas analysis was deferred since the SpO_2_ was within the expected range for a lifelong resident at 4,100 m and did not indicate severe hypoxemia, the clinical priority was managing the acute encephalopathy. He was hypertensive (144/92 mmHg) and exhibited plethoric facies with pronounced cyanosis of the nail beds, lips, and oral mucosa—findings more severe than those in his healthy cohabiting relatives (Figure 1). He was confused (Glasgow Coma Scale 13), with dysarthric speech and partial compliance with simple commands, consistent with higher cortical dysfunction. Spontaneous limb movements were preserved with normal muscle tone, and no pathological reflexes or meningeal signs were present.

Laboratory investigations revealed leukocytosis (13.40 × 10^9^/L) with neutrophilic predominance (88.8%), extreme erythrocytosis (hemoglobin 229 g/L, hematocrit 77.4%), and elevated high-sensitivity C-reactive protein (69.72 mg/L). Coagulation studies showed prolonged prothrombin time (18.5 s) with normal fibrinogen (4.49 g/L). Metabolic abnormalities included mild hyperhomocysteinemia (19.15 μmol/L), unconjugated hyperbilirubinemia (total bilirubin 93.3 μmol/L, indirect fraction 68.9 μmol/L), and severe hyperuricemia (1,195 μmol/L). Lumbar puncture yielded an opening pressure of 240 mmH_2_O with normal cerebrospinal fluid (CSF) analysis. Serum EPO was paradoxically suppressed at 1.2 mIU/mL (reference range: 5.44–26.25)—a finding contrary to the expected altitude-driven EPO elevation in secondary erythrocytosis. Autoimmune and antiphospholipid antibody panels were negative.

**Figure 2:**
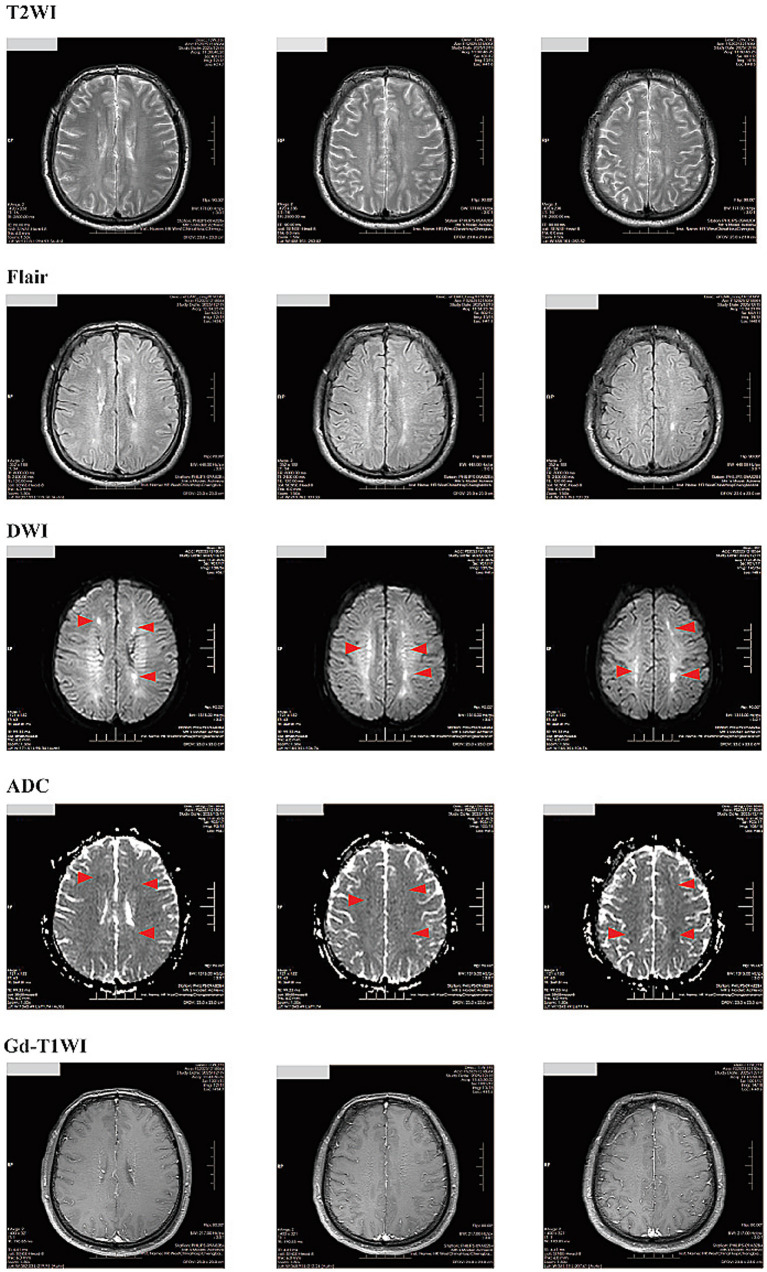
Cranial neuroimaging findings. This figure presents the patient’s cranial magnetic resonance imaging (MRI) scans, including T2-weighted imaging (T2WI), Fluid-Attenuated Inversion Recovery (FLAIR), Diffusion-Weighted Imaging (DWI), Apparent Diffusion Coefficient (ADC), and Gadolinium-enhanced T1-weighted imaging (Gd-T1WI). Multiple punctate and small patchy lesions exhibiting restricted diffusion are evident within the bilateral frontoparietal lobes and centrum semiovale, as indicated by the red arrows. No contrast enhancement was observed on post-gadolinium sequences.

**Figure 3:**
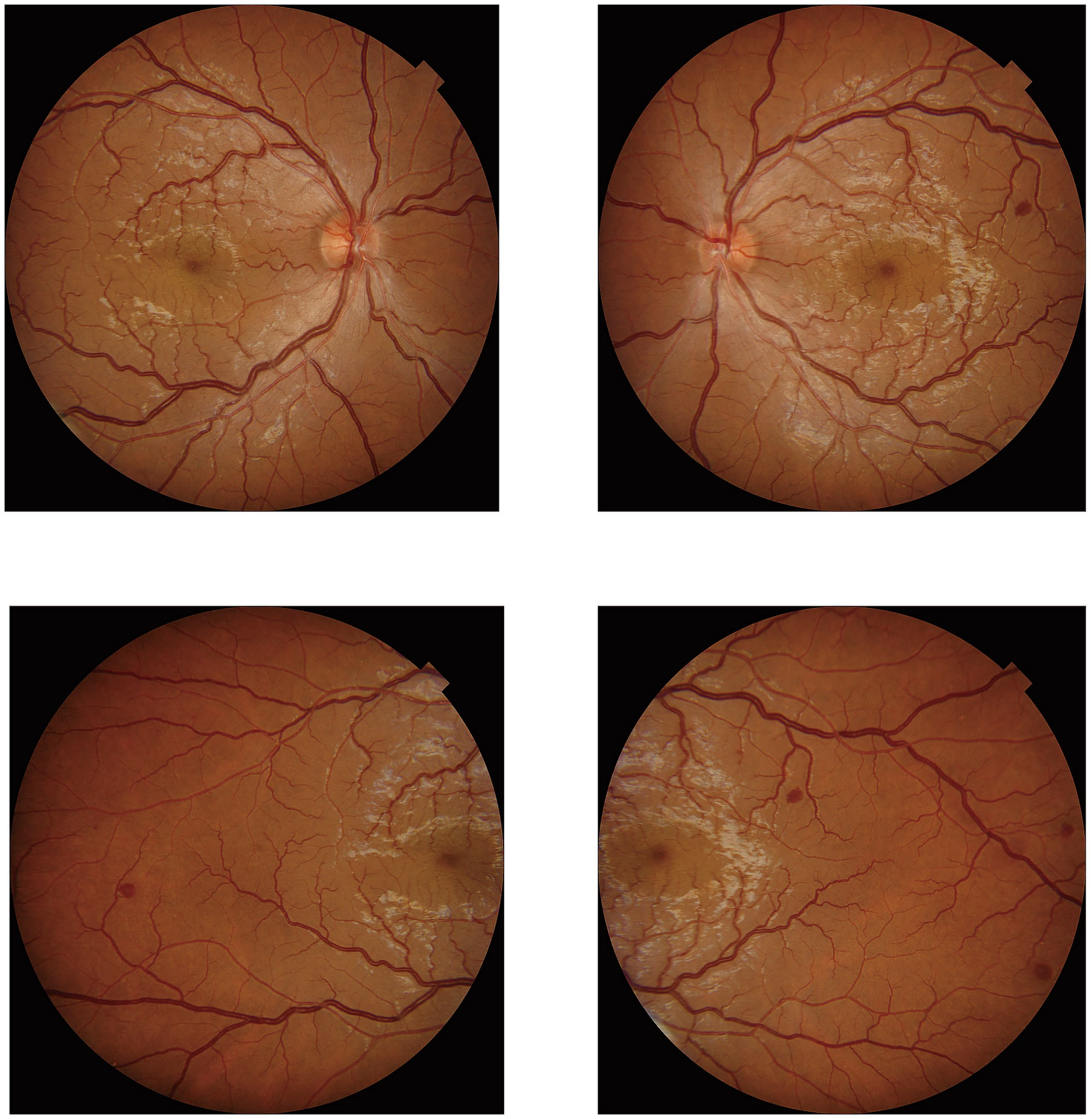
Fundus photography. The images reveal retinal venous tortuosity and dilation in both eyes, accompanied by discrete punctate and patchy hemorrhagic foci.

Pre-admission chest computed tomography (CT) showed pulmonary inflammatory infiltrates. Cranial CT on admission revealed diffuse cerebral edema. Brain MRI with DWI detected multiple punctate and patchy diffusion-restricted lesions in the bilateral frontoparietal lobes and centrum semiovale, without post-contrast enhancement (Figure 2). MR venography excluded cerebral venous sinus thrombosis. Echocardiography showed right ventricular enlargement with pulmonary artery dilation. Abdominal ultrasonography revealed cholelithiasis and mild splenomegaly (intercostal thickness 5.6 cm). Fundoscopic examination showed retinal venous tortuosity and hemorrhagic foci (Figure 3). Cerebral angiography and ambulatory ECG monitoring were unremarkable.

### Clinical course

**Figure 4:**
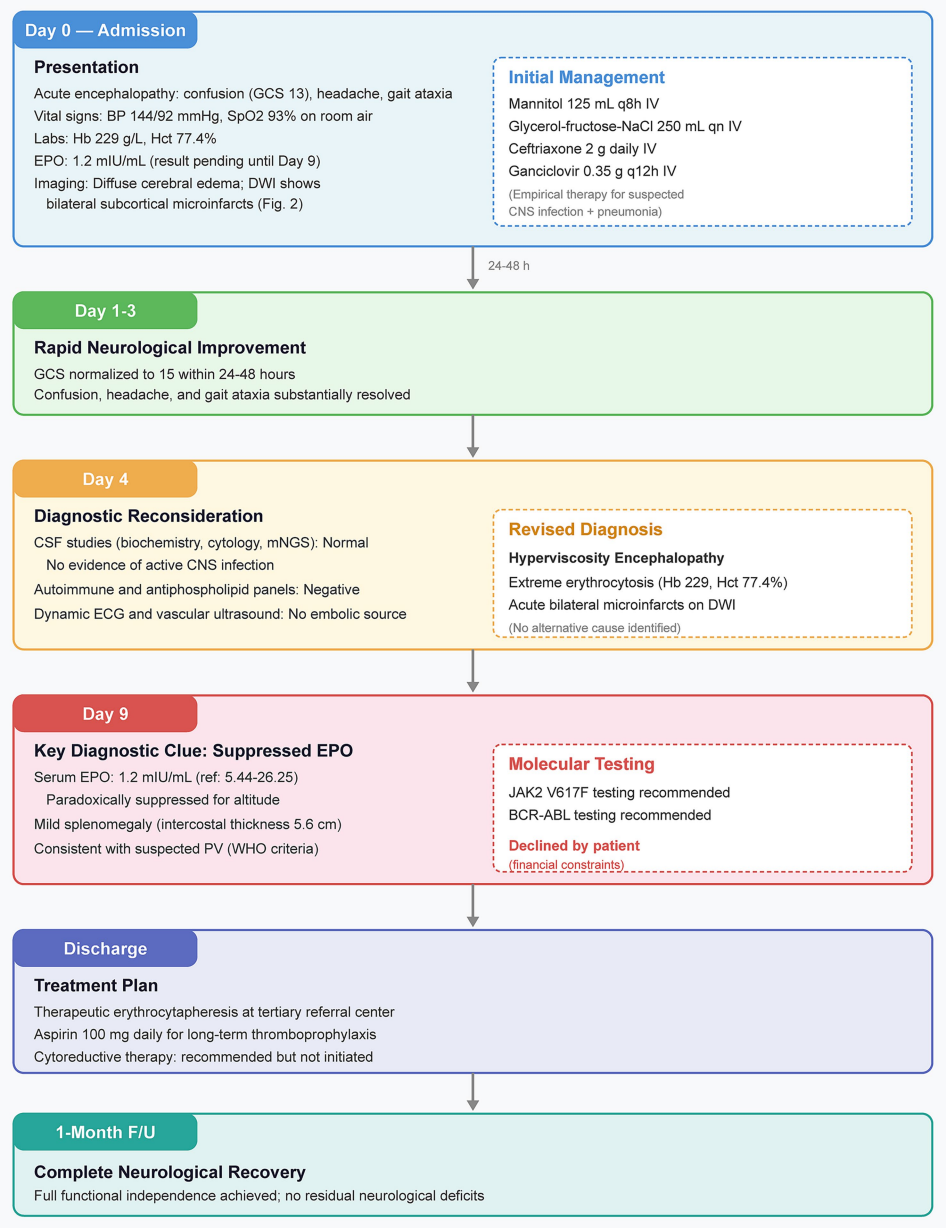
Clinical course and management timeline. CNS, central nervous system; CSF, cerebrospinal fluid; DWI, diffusion-weighted imaging; EPO, erythropoietin; F/U, follow-up; GCS, Glasgow Coma Scale; mNGS, metagenomic next-generation sequencing; PV, polycythemia vera.

The patient’s management evolved dynamically over the hospitalization period (summarized in Figure 4). The patient initially received empirical therapy for suspected intracranial infection with concomitant pneumonia: intravenous mannitol (125 mL every 8 h) and glycerol-fructose-sodium chloride (250 mL nightly) for intracranial hypertension; ceftriaxone (2 g daily) for antibacterial coverage; and ganciclovir (0.35 g every 12 h) for antiviral prophylaxis. Neurological symptoms improved substantially within 24–48 h, and the Glasgow Coma Scale normalized to 15.

On day 4, normal CSF studies and the neuroimaging pattern prompted reclassification to hyper-viscosity encephalopathy. The triad of acute encephalopathy, extreme erythrocytosis, and chronic high-altitude exposure initially suggested high-altitude secondary polycythemia with cerebral infarction. However, the absence of similar findings in genetically related cohabitants raised suspicion for PV. This hypothesis was strengthened on day 9 by the finding of markedly suppressed EPO (1.2 mIU/mL). Hematology consultants recommended molecular testing for JAK2 V617F and BCR-ABL, but the patient declined, citing financial constraints. He chose instead to undergo therapeutic erythrocytapheresis at a referral center and began aspirin (100 mg daily) for thromboprophylaxis. At one-month follow-up, he had made a complete neurological recovery with full functional independence.

## Discussion

This case illustrates the difficulty of diagnosing suspected PV at extreme high altitude. PV, the most common myeloproliferative neoplasm (MPN), is defined by autonomous erythroid proliferation often accompanied by leukocytosis and thrombocytosis (9). Its neurological complications are dominated by thrombotic events, including cerebral infarction (2, 3), cerebral venous sinus thrombosis (4, 5), and hemorrhagic phenomena (7).

The current World Health Organization diagnostic framework (1) mandates fulfillment of specific criteria: major criteria include (1) hemoglobin >16.5 g/dL in men or >16.0 g/dL in women (equivalently, hematocrit >49% or >48%), (2) hypercellular bone marrow with pleomorphic megakaryocytes, and (3) presence of JAK2 V617F or exon 12 mutations. A subnormal serum EPO level constitutes the minor criterion. Notably, bone marrow examination may be omitted when major criterion 3 and the minor criterion coexist with hemoglobin exceeding 18.5 g/dL in men or 16.5 g/dL in women. Our patient had markedly elevated hemoglobin (22.9 g/dL) and hematocrit (77.4%) with suppressed EPO (1.2 mIU/mL), but molecular confirmation was not possible because the patient declined JAK2 testing.

The clinical picture was consistent with a hyperviscosity/thrombotic state: extreme erythrocytosis (hemoglobin 229 g/L, hematocrit 77.4%), elevated fibrinogen (4.49 g/L), and multiple acute subcortical micro-infarcts on DWI (Figure 2), without venous sinus thrombosis. Alternative diagnoses were systematically excluded. CSF studies (routine biochemistry, cytology, and metagenomic next-generation sequencing) showed no evidence of active central nervous system (CNS) infection. Autoimmune and antiphospholipid panels were negative. Dynamic ECG and vascular ultrasound identified no cardiogenic or large-artery embolic source. The rapid improvement within 24 h of osmotic therapy argued against an inflammatory or autoimmune CNS disorder.

The key diagnostic clue was the suppressed EPO level (1.2 mIU/mL). The assay was performed on a chemi-luminescence platform (MAGLUMI X8, reference range: 5.44–26.25 mIU/mL). We note that the diagnostic threshold of 3.1 mIU/mL (10) was derived from a different immunoassay platform; inter-assay variability should therefore be considered. In secondary polycythemia, EPO is typically elevated in response to hypoxia (11); a suppressed level therefore raised suspicion of a clonal disorder. Mild splenomegaly (intercostal thickness 5.6 cm) further supported this impression. A thorough history excluded secondary erythrocytosis from chronic obstructive pulmonary disease, congenital heart disease, tobacco use, or medications (Sodium-Glucose co-Transporter-2 inhibitors, androgens).

Chronic mountain sickness (CMS; Monge disease) is a key differential diagnosis in high-altitude natives presenting with extreme erythrocytosis, as it shares features such as elevated hemoglobin, cyanosis, and headache (12, 13). The Qinghai CMS score was not formally assessed at presentation because of the patient’s acute encephalopathic state. However, several findings argue against CMS as the primary diagnosis in this case: (1) peripheral oxygen saturation was 93% on room air, whereas CMS is characterized by severe hypoxemia (SpO_2_ typically <85%) (12, 14); (2) serum EPO was suppressed (1.2 mIU/mL), whereas in CMS the EPO axis remains responsive to hypoxia and EPO levels are normal or appropriately elevated (8, 15); and (3) the multifocal diffusion-restricted lesions on brain MRI are not typical of CMS, which has been associated with vasogenic cerebral edema rather than acute micro-infarcts (16). Nevertheless, prospective use of the CMS scoring system is recommended when evaluating high-altitude residents with unexplained erythrocytosis.

Second, this case may illustrate a multiple-hit model of thrombogenesis in suspected PV. The preceding upper respiratory infection may have contributed through several mechanisms: (1) inflammatory cytokine release worsening pre-existing endothelial injury (17); (2) dehydration-induced hemoconcentration; and (3) blood pressure fluctuations disrupting cerebral autoregulation (18). Together, these factors may have triggered acute decompensation (19), leading to widespread acute micro-infarcts and vasogenic edema. This sequence could explain the rapid response to anti-infective and osmotic therapy.

Several additional laboratory abnormalities warrant comment. Unconjugated hyperbilirubinemia (68.9 μmol/L) was present with a mildly elevated lactate dehydrogenase (LDH, 322 U/L; reference range 120–250 U/L), while the direct Coombs test was negative. Reticulocyte counts and haptoglobin were not measured. The mildly elevated LDH could be attributed to either ineffective erythropoiesis or low-grade hemolysis, but the negative Coombs test, the absence of anemia, and the extreme erythrocytosis together favor ineffective erythropoiesis—a known feature of MPNs (20, 21)—as the primary mechanism. Future cases should include haptoglobin and reticulocyte counts to clarify this distinction. The prolonged prothrombin time (PT, 18.5 s) with normal fibrinogen likely represents a transient coagulopathy of uncertain significance; no bleeding occurred, and the PT normalized on follow-up. Mild hyperhomocysteinemia (19.15 μmol/L) has been reported in patients with PV and other MPNs (22, 23) and is a recognized risk factor for both venous and arterial thrombosis (24), which may have contributed to the prothrombotic state in this patient. BCR-ABL testing was included in the MPN screening panel to exclude chronic myeloid leukemia, as concurrent testing for BCR-ABL and JAK2 V617F is part of the initial molecular workup for suspected MPN (25, 26), although it is not routinely indicated when PV is the primary clinical suspicion.

The therapeutic approach followed a stepwise hierarchy: emergency intracranial pressure control (mannitol and glycerol-fructose for intracranial pressure control), followed by definitive hematologic management (therapeutic erythrocytapheresis), and then long-term thromboprophylaxis (daily aspirin for thromboprophylaxis) with consideration of cytoreductive therapy. This escalation is important for optimizing outcomes in acute hyperviscosity emergencies.

The main diagnostic challenge in high-altitude settings is the tendency to attribute all hematologic abnormalities to environmental hypoxia (8). This anchoring bias can lead to premature closure with a diagnosis of “high-altitude secondary polycythemia complicated by cerebral infarction.” The suppressed EPO—contrary to the expected hypoxic response—pointed instead to autonomous, EPO-independent erythropoiesis (11). We therefore suggest that serum EPO measurement be included in the initial workup of any high-altitude resident with unexplained erythrocytosis or thrombosis.

Several limitations should be acknowledged. The absence of bone marrow biopsy and molecular confirmation (JAK2 V617F, BCR-ABL) precludes a definitive diagnosis of PV and does not fully exclude congenital polycythemias (27). Nevertheless, the observed EPO level (1.2 mIU/mL) is well below the diagnostic threshold of 3.1 mIU/mL, which carries 80.5% sensitivity and 87.8% specificity for PV (10). In summary, this case underscores that geographical context should not replace rigorous diagnostic investigation. For high-altitude residents with unexplained erythrocytosis, systematic EPO measurement followed by JAK2 mutation analysis should be considered to identify underlying clonal disorders and to guide appropriate treatment and long-term risk reduction.
